# Macronutrient Intake Influences the Effect of 25-Hydroxy-Vitamin D Status on Metabolic Syndrome Outcomes in African American Girls

**DOI:** 10.1155/2012/581432

**Published:** 2012-06-26

**Authors:** Anna L. Newton, Lynae J. Hanks, Ambika P. Ashraf, Elizabeth Williams, Michelle Davis, Krista Casazza

**Affiliations:** ^1^Department of Nutrition Sciences, University of Alabama at Birmingham, 1675 University Boulevard, WEBB 439 Birmingham, AL 35294-3360, USA; ^2^Division of Pediatric Endocrinology, Department of Medicine, University of Alabama at Birmingham, Birmingham, AL 35294-3360, USA

## Abstract

The objectives were to determine the effect of macronutrient modification on vitamin D status and if change in 25-hydroxy-vitamin D concentration influences components of metabolic syndrome in obese African American girls. *Methods*. Five-week intervention using reduced CHO (43% carbohydrate; 27% fat: SPEC) versus standard CHO (55% carbohydrate; 40% fat: STAN) eucaloric diet. Subjects were 28 obese African American females, aged 9–14 years. Dual energy X-ray absorptiometry and meal test were performed at baseline and five weeks. *Results*. Approximately 30% of girls had metabolic syndrome. Serum 25OHD increased in both groups at five weeks [STAN: 20.3 ± 1.1 to 22.4 ± 1.1 (*P* < 0.05) versus SPEC: 16.1 ± 1.0 to 16.8 ± 1.0 (*P* = 0.05)]. The STAN group, increased 25OHD concentration over five weeks (*P* < 0.05), which was positively related to triglycerides (*P* < 0.001) and inversely associated with total cholesterol (*P* < 0.001) and LDL (*P* < 0.001). The SPEC group, had increase in 25OHD (*P* = 0.05), which was positively related to fasting insulin (*P* < 0.001) and insulin sensitivity while inversely associated with fasting glucose (*P* < 0.05). The contribution of vitamin D status to metabolic syndrome parameters differs according to macronutrient intake. Improvement in 25OHD may improve fasting glucose, insulin sensitivity, and LDL; however, macronutrient intake warrants consideration.

## 1. Introduction

The steady rise in prevalence of pediatric obesity over the past three decades has been accompanied by accumulation of risk factors for metabolic syndrome (MetSyn) in childhood and adolescence. The occurrence of hypovitaminosis D (expressed as levels <20 ng/mL of circulating 25-hydroxy vitamin D (25OHD)) has been increasingly documented in the same population [1, 2]. Moreover, children/adolescents with hypovitaminosis D have been reported to experience greater instances of hypertension, hypertriglyceridemia, hyperglycemia, and low high-density lipoprotein cholesterol (HDL) [1, 3, 4]. Further, it has been proposed that elevated parathyroid hormone (PTH), consequential to chronic low vitamin D level, is mechanistically involved in the adverse perturbations of risk factors underlying MetSyn [5]. Given the emerging identification of vitamin D as an integral player in numerous metabolic pathways, it stands to reason that vitamin D status in the pediatric populace may play a role in the prevalence of metabolic disease risk factors [6, 7]. 

The relationship between 25OHD status and metabolic health is not equally distributed across groups. In particular, the relationship is more apparent among African American (AA) females, particularly those who are overweight/obese [8–11]. Although greater prevalence of hypovitaminosis D among obese AA may be in part attributed to skin pigmentation and sequestering of vitamin D in adipose tissue, differences in classical endocrine effects (e.g., PTH and insulin response) likely also play a role [11]. Further, diet may modify the relationship between vitamin D bioavailability and underlying metabolic pathways. It is known that 25OHD level is dependent on intestinal absorption of dietary vitamin D, the extent to which vitamin D may exert effects on metabolic factors is at least in part dependent upon macronutrient profile of the diet. The metabolic response to dietary composition, specifically carbohydrate quantity of a meal, influences the postprandial cascade of events (increased glucose, insulin, lipogenesis, glycogenesis, etc.). Vitamin D has been independently associated with these processes, and the fact that vitamin D insufficiency stimulates secretion of PTH cannot be ignored. Data from this group has demonstrated that the physiological response to macronutrient concentration of the diet differs among racial groups [12]; however, to our knowledge there has been no investigation regarding the contribution of macronutrient profile to vitamin D involvement with metabolic components. Accordingly, the independent and interactive contribution of diet and vitamin D status (and consequent PTH level) on metabolic risk warrants investigation, particularly among obese adolescents.

Understanding the extent to which macronutrient composition influences vitamin D bioavailability may be particularly important during growth and development. Adolescence characterizes a time when risk factors for MetSyn can be identified, and modification of the dietary profile represents a strategy in which intervention may have the greatest directive impact. Therefore, the objective of this study was to determine the effect of macronutrient modifications on vitamin D status and if change in 25OHD concentration would influence components of MetSyn in obese AA girls. Further, as AA females are a population group that are at increased metabolic risk, this study seeks to evaluate the effect of macronutrient modification on associations of vitamin D concentrations and potential influence of PTH in AA adolescent females to parameters of MetSyn (fat distribution, insulin sensitivity, glucose tolerance, lipid concentrations, and blood pressure).

## 2. Methods

Participants included 28 overweight/obese AA girls aged 9–14 years. Obesity was defined as greater than 95th sex- and age-specific body mass index (BMI) percentile, and exclusion criteria were medical diagnosis and/or taking medications known to affect body composition, metabolism, and cardiac function. Participants were recruited through newspaper advertisements, flyers posted at various community partnerships, and by word-of-mouth. The nature, purpose, and possible risks of the study were carefully explained to each participant and guardian(s), and informed assent and consent, respectively were obtained. The protocol was approved by the Institutional Review Board for human subjects at the University of Alabama at Birmingham (UAB). All measurements were performed at the Participant and Clinical Interactions Resources (PCIRs) and the Department of Nutrition Sciences at UAB between 2008 and 2009.

### 2.1. Protocol

This study was part of a 16-week intervention comparing the effectiveness of a reduced CHO (43% carbohydrate: SPEC) versus a standard CHO (55% carbohydrate: STAN) diet on weight loss and metabolic health. Included data were derived from the initial five-week eucaloric phase, during which time the goal was for participants to maintain their baseline weight while consuming respective diets. Participants were block-randomized to one of the two diets which they remained on for the duration of the study. All food was provided, with the amount determined according to calculated individual needs determined by resting energy expenditure (REE; assessed via indirect calorimetry) multiplied by a 1.2 activity factor (averaging about 2000 kcal/d).

At baseline, participants attended two visits. The first visit entailed a physical examination by the study pediatrician, questionnaires on typical diet and regular physical activity, and a full-body dual energy X-ray absorptiometry (DXA) scan. At the second visit, participants reported to the PCIR in the morning in the fasted state for metabolic testing. After 30 minutes of rest, REE was assessed, and a liquid mixed-meal test (LMMT) was performed. To ensure weight stability, participants were weighed twice per week at food pickup to evaluate the caloric prescription throughout the five-week eucaloric phase. Weight changes exceeding two kilograms from baseline resulted in caloric modification in effort to maintain weight. Participants were asked to maintain their regular level of physical activity. At the duration of five weeks, the DXA scan, indirect calorimetry, and LMMT were repeated.

### 2.2. Diets

The SPEC diet comprised 42% of energy from CHO and 40% of energy from fat, whereas the STAN diet comprised 55% of energy from CHO and 27% of energy from fat. Both diets contained a similar content of protein (about 18%). Baseline energy levels for all diets were 1600 kcal with the addition of 100 to 400 kcal snack increments to participants who required more than 1600 kcal per day. All diets included culturally- and age-appropriate foods with no inclusion of supplements or formulas, and noncaloric fluid intake of ≥64 fluid ounces per day was recommended. All meals were prepared and packaged in the research kitchen at the UAB PCIR. A trained registered dietitian coded and entered data from each diet into the computerized Nutrition Data System for Research (NDSR).

### 2.3. Anthropometrics

The same registered dietitian obtained anthropometric measurements for all participants. Participants were weighed (Scale-Tronix 6702W; Scale-Tronix, Carol Stream, IL, USA) to the nearest 0.1 kg in minimal clothing without shoes. Height was also recorded without shoes using a digital stadiometer (Heightronic 235; Measurement Concepts, Snoqualmie, WA, USA). BMI percentile and obesity status (≥95th percentile) was calculated using sex- and age-specific CDC growth charts based on these measurements (http://apps.nccd.cdc.gov/dnpabmi/). 

### 2.4. Body Composition and Fat Mass Index

Body composition was measured by DXA using GE Lunar Prodigy densitometer (GE LUNAR Radiation Corp., Madison, WI, USA). Participants were scanned in light clothing, while lying flat on their backs with arms at their sides. Due to size limitations, participants not fitting within the scanning box were right-sided hemiscanned with the left side estimated as per instrument protocol. Fat mass index calculated as total body fat divided by height was used as a covariate [13].

### 2.5. Liquid Mixed Meal Test/Insulin Sensitivity

Insulin response to a standardized meal was determined from an index of insulin sensitivity and secretion through frequent blood sampling following ingestion of a LMMT (Carnation Instant Breakfast prepared with whole milk). To perform the LMMT, a flexible intravenous catheter was placed in the antecubital space of the left arm. The “dose” for the liquid meal test was obtained according to amount of lean body mass (LBM) of the participant (1.75 g CHO/kg LBM). Participants were required to consume the meal within five minutes. Blood was drawn at baseline (three samples over 15 minutes) prior to initiation of meal consumption at time “zero.” Subsequent blood samples were drawn every five minutes from time zero to 30 minutes, every 10 minutes from time 40 to 180 minutes, and at 210 and 240 minutes. Using the obtained measures, the insulin sensitivity index (SI) was calculated by oral glucose model [14].

### 2.6. Assay of Metabolites

Glucose was measured in 12 *μ*L sera with the glucose oxidase method using a SIRRUS analyzer (interassay CV 2.56%). Insulin was analyzed using a TOSOH 1800 Automated Immunoassay Analyzer. Assay sensitivity is 15.42 pmol/L, mean intraassay CV is 4.69%, and interassay CV is 6.0%. Triglycerides (TGs) were assessed with the glyceryl phosphate method. HDL was analyzed using a two-reagent system involving stabilization of low-density lipoprotein cholesterol (LDL), very low-density lipoprotein cholesterol (VLDL), and chylomicrons using cyclodextrin and dextrin sulfate, and subsequent enzymatic-colorimetric detection of HDL. 25OHD and PTH concentration was obtained from fasting sera drawn and assayed in the UAB Core Laboratory with liquid chromatography/tandem mass spectrometry technique and a two-site radiometric assay, respectively. 

### 2.7. Statistical Analysis

Differences at baseline in descriptive characteristics between girls in the two diet groups were examined using *t*-tests. The differences between diet groups regarding metabolic parameters were evaluated using ANOVA to allow for inclusion of covariates (pubertal stage, fat mass index, and baseline measures). Multivariate linear regression (Model A) was used to analyze the contribution of the change in 25OHD concentration over five weeks to individual components of metabolic syndrome. Due to the intricate relationship with PTH, for each component that was observed to be associated with 25OHD, a second regression model (Model B) was analyzed with inclusion of PTH as a covariate. To conform to the assumptions of linear regression, all statistical models were evaluated for residual normality and logarithmic transformations were performed when appropriate. All data were analyzed using SAS 9.2 software. Subsequently, models were evaluated by presence or absence of MetSyn. MetSyn was defined as meeting the IDF criteria for at least three of the components and was coded as zero for absence and one for presence. In addition, interaction terms were created (diet by change in 25OHD) and included in regression models to test the moderation by diet and change in 25OHD of relationships between metabolic outcomes.

## 3. Results

**Figure 1:**
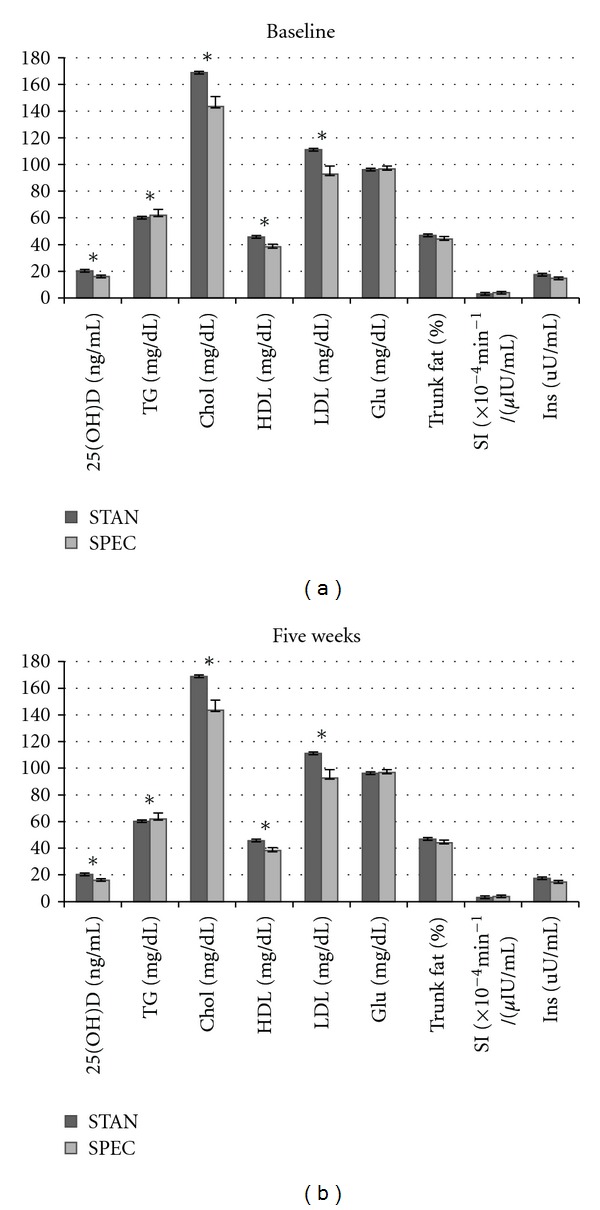
Components of metabolic syndrome at baseline and five weeks. *Indicates significant difference between diet groups (*P* < 0.5). Abbreviations: circulating vitamin D (25OHD), triglycerides (TGs), cholesterol (Chol), high-density lipoprotein (HDL), low-density lipoprotein (LDL), fasting glucose (Glu), insulin sensitivity (SI), and insulin (Ins).

Descriptive characteristics and body composition at baseline in the total sample and by diet group are presented in Table 1. There were no differences between groups across variables, with the exception of percent body fat, which was significantly higher in the STAN diet group versus the SPEC diet group. At baseline, 29% of participants met the criteria for MetSyn, and individual components are illustrated at baseline and five weeks in Figure 1. Differences between diet groups were observed at baseline for circulating 25OHD and all lipid parameters [TG, LDL, HDL, and total cholesterol (tot chol)], and at five weeks for circulating 25OHD and TG.

**Figure 2:**
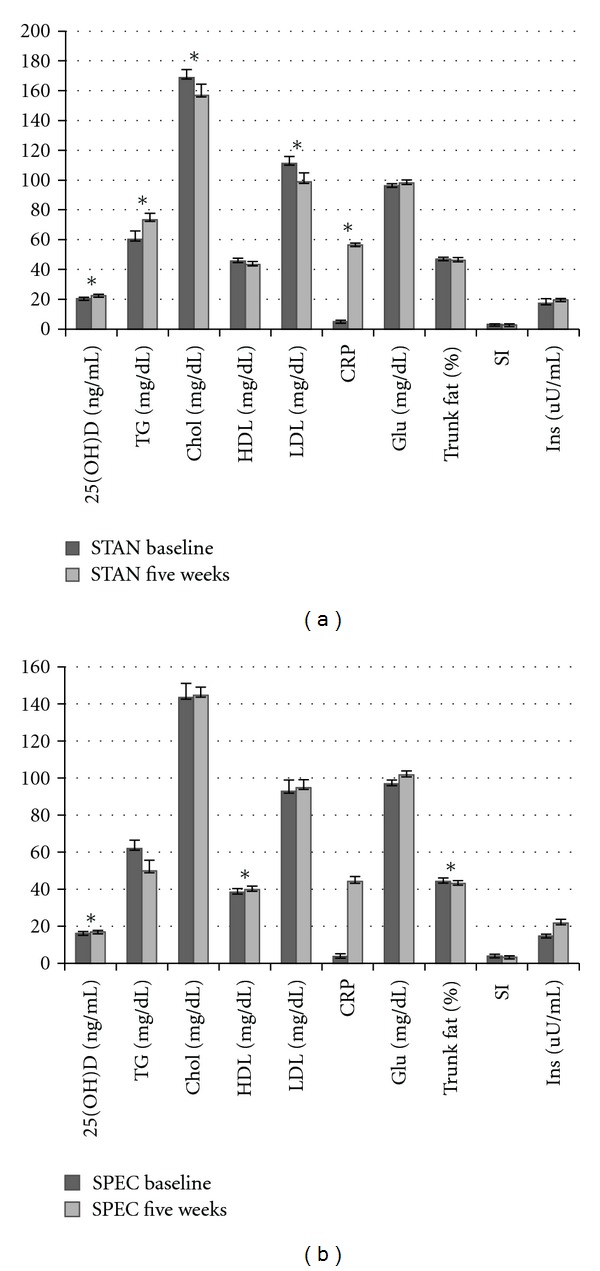
Diet group evaluations related to MetSyn components. *Indicates significant differences between baseline and five weeks (*P* < 0.05). Abbreviations: circulating vitamin D (25OHD), triglycerides (TGs), cholesterol (Chol), high-density lipoprotein (HDL), low-density lipoprotein (LDL), fasting glucose (Glu), insulin sensitivity (SI), and insulin (Ins).

Diet group evaluations related to MetSyn components are illustrated in Figure 2. Among those consuming the STAN diet, significant differences were observed from baseline to five weeks for circulating 25OHD (*P* < 0.05) and all lipid parameters (*P* < 0.001), except HDL. A significant increase was revealed for circulating 25OHD and TG with a decrease in total chol and LDL across the five-week period. Among those consuming the SPEC diet, a significant increase in circulating 25OHD and insulin (*P* = 0.05 and *P* < 0.001, resp.) from baseline to five weeks was distinguished. Although an increase in circulating 25OHD was observed for both diet groups, circulating 25OHD levels were lower at baseline in participants presenting with MetSyn versus those with no MetSyn (Table 2). Similarly, greater PTH concentrations were observed in those presenting with MetSyn than those without MetSyn at both baseline and five weeks (*P* < 0.001). Further, individuals meeting criteria for MetSyn displayed no increase in circulating 25OHD across the five-week study period unlike participants without MetSyn. 

The contribution of change in circulating 25OHD (ΔOHD) over the five weeks to MetSyn components is presented in Table 3. Two models were used to assess the relationship. Model A illustrates the independent contribution of ΔOHD to metabolic parameters, and Model B presents the inclusion of both ΔOHD and PTH as independent variables. Among those consuming the STAN diet, an inverse relationship between ΔOHD and LDL (*P* < 0.05) was observed (Model A); in Model B, the association remained significant (*P* < 0.05). However, in this model a marginal association between ΔOHD and fasting glucose (*P* = 0.07) was observed as well as an independent contribution of PTH to fasting glucose concentration (*P* < 0.001). Additionally, a significant positive association between PTH and TG (*P* < 0.01) was observed. Among those consuming the SPEC diet, ΔOHD (Model A) was inversely associated with fasting glucose (*P* < 0.05) and positively associated with SI (*P* < 0.05); in Model B, these relationships remained, and an inverse relationship between PTH and SI was observed, whereas there was no relationship between PTH and fasting glucose.

**Figure 3:**
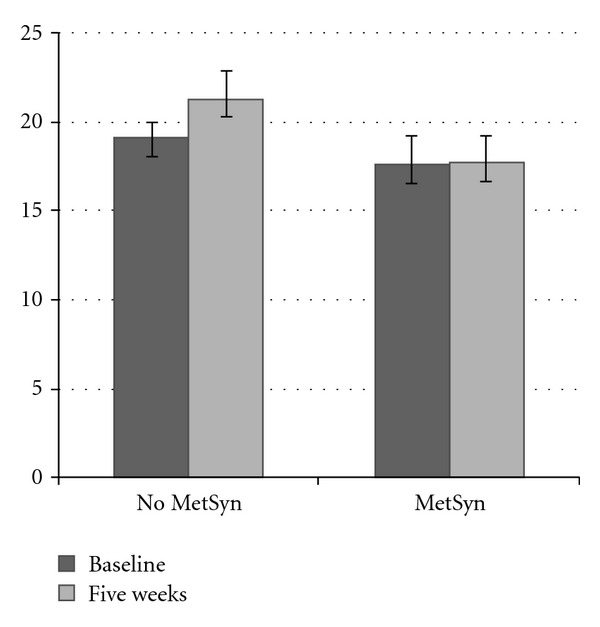
Circulating 25OHD levels at baseline and five weeks in the presence or absence of MetSyn.

At five weeks, 32% of participants met the criteria for MetSyn. There was no difference in those meeting criteria between diet groups; however, when the interaction term (ΔOHD × diet) was evaluated, those individuals who presented with MetSyn at five weeks displayed significantly positive associations between the interaction term and TG (*P* < 0.001), HDL (*P* < 0.01), LDL (*P* < 0.01), and systolic blood pressure (SysBP; *P* < 0.01). SI and insulin were inversely associated with the interaction term (*P* < 0.01) in those individuals who did not present with MetSyn at five weeks. After five weeks, there was an increase in circulating 25OHD among those without MetSyn, yet no change was observed in those with MetSyn (Figure 3). Further, 25OHD concentration among those with MetSyn was at a level that would be deemed insufficient based on currently accepted criteria [15].

## 4. Discussion

Vitamin D's emerging role as an integral component of metabolism is accompanied by the occurrence of risk factors for metabolic disease early in life and displays a critical conduit to which dietary intervention may facilitate improved metabolic outcomes. The objective of this study was to determine the effect of macronutrient modifications in the absence of weight loss on vitamin D status and if ΔOHD concentration would influence components of MetSyn in AA adolescent females. For those consuming a reduced carbohydrate diet, ΔOHD was inversely associated with fasting glucose and positively associated with SI. These relationships were maintained with inclusion of PTH. Clustering of metabolic parameters occurred such that glucose-related parameters in those without MetSyn, and lipid-related components in those with MetSyn were significantly associated with an interaction of diet and ΔOHD. These results provide some evidence regarding the role that vitamin D may exert on alterations in the biological response to macronutrients lending itself to further exploration.

Numerous studies in children have suggested metabolic effects of 25OHD on several markers of glucose (e.g., fasting glucose, insulin concentrations, and HOMA score) [16–19] and lipid metabolism (e.g., TG, HDL, and LDL) [18, 19]. In this sample, the interaction term (diet and ΔOHD) was positively associated with lipid profile among those meeting the criteria for MetSyn. Similarly, an independent effect of vitamin D on lipid profile was suggested by a weight-loss intervention study that found vitamin D supplementation resulted in improved lipid profile in the supplementation group despite similar weight-loss in nonsupplemented group [20]. Additionally, Al-Daghri and colleagues observed an association between total cholesterol and LDL and 25OHD which was apparent among adults with Type 2 Diabetes, but not in those without. Further, there was a reversal of MetSyn manifestations with vitamin D status correction [21]. From a physiologic standpoint, metabolic response to diet and potential relation with vitamin D status is an area in need of further exploration. The differential impact of the interaction between macronutrient composition and ΔOHD is of particular interest in light of the lack of consensus regarding optimal 25OHD concentration as it relates to vitamin D recommendations.

It has been suggested that a reduction in carbohydrate intake requires increased insulin resistance to maintain glucose homeostasis, particularly during reproductive development [22]. Among the SPEC diet group, glucose concentration increased to a greater extent in those with a lesser increase in 25OHD, respectively insulin sensitivity decreased to a greater extent in those with a lesser increase in 25OHD.It is plausible that vitamin D mediates the effect of reduced carbohydrate intake through its direct action on pancreatic *β*-cell function [23]. Conversely, in the STAN diet group, in which carbohydrate intake reflected that which is more typical of the adolescent population [24], manifestations of altered 25OHD concentration were apparent in lipid parameters. LDL concentration increased to a greater extent in those with a lesser increase in 25OHD. Although cross-sectional analysis has consistently identified a favorable effect of 25OHD concentration on LDL, vitamin D's function in lipid metabolism remains uncertain. The confluence of macronutrient composition adds further complexity. The relationship observed in this study is clear, and our findings suggest that effect of dietary composition on 25OHD bioavailability warrant consideration.

Establishing the role of vitamin D in relation to MetSyn is complicated by its reciprocal association with PTH; in addition, several of the proposed predictors of MetSyn are also known to be associated with PTH. Not surprisingly, in those meeting criteria, as opposed to those not meeting the criteria for MetSyn, PTH concentrations were significantly greater at both baseline and five weeks of this study. Many [25–27], but not all [28], studies report an inverse relationship between 25OHD and PTH. Moreover, AA generally present with lower 25OHD concentrations and higher PTH relative to European American counterparts [16, 26, 29, 30]. The relationship between vitamin D and PTH is influenced by various factors during growth and development, including dietary macronutrient composition, supported by findings reported herein. Independent of the positive association with vitamin D, an inverse relationship was found between PTH and SI, only apparent in those consuming the reduced carbohydrate, specialized diet. This may be translated into independent pathways of both PTH and vitamin D in linkage with MetSyn. It has been recently reported that PTH concentration, but not 25OHD, contributed to MetSyn in obese adults [5]. Support is provided for the postulation that influence by each of these factors diverges according to dietary composition.

This study had many strengths as well as evident limitations. Comprehensive phenotyping using robust measures of body composition is a major strength. The provision of food to each participant is an additional advantage because it ensured some degree of dietary control. Despite these strengths, it is important to evaluate certain shortcomings of this investigation. In noninstitutionalized subjects, dietary adherence is difficult to ascertain; beyond monitoring for weight change, the consumption of additional foods other than those provided cannot be certain. Also, the modest sample size and inclusion of only one racial group limits generalizability to other populations. Each participant was in the >99% BMI percentile, which may limit the ability to detect changes in outcome measures across body habitus. Finally, it is important to note that the short duration of the intervention may limit ability to identify the long-term effects of the diet on vitamin D.

## 5. Conclusions

Initiation and progression to MetSyn encompasses perturbations in glucose and lipid metabolism and is exacerbated by overweight/obesity, with AA females experiencing disproportionate incidence. Similar to what has been reported in other studies [7, 30–32], 29% of girls in this study presented with MetSyn and 25OHD was observed to be significantly lower and deemed insufficient in those presenting with MetSyn.  Additionally, our results support the effect of macronutrient composition on the contribution of circulating 25OHD to parameters associated with MetSyn. Our findings also indicate that improvement in circulating 25OHD concentrations may normalize glucose parameters associated with initiation and progression to MetSyn. Additionally, though the mechanistic response of PTH to diet is unclear, its reciprocal relationship with vitamin D may mediate the effects of a reduced carbohydrate diet. This investigation builds upon previous findings which imply unique metabolic characteristics of peripubertal AA females. In this context and considering the consistent reports of vitamin D insufficiency among this group, our findings may help inform recommendation efforts.

## Figures and Tables

**Table 1:** Baseline descriptive characteristics.

	Total	STAN	SPEC
	(*n* = 28)	(*n* = 15)	(*n* = 13)
Age (yrs)	12.4 ± 0.3	12.5 ± 0.4	12.4 ± 0.5
Tanner stage	4.4 ± 0.2	4.4 ± 0.3	4.4 ± 0.3
Height (cm)	159.5 ± 1.5	159.3 ± 2.0	160.3 ± 2.3
Weight (kg)	89.6 ± 4.6	93.8 ± 7.5	85.1 ± 4.8
BMI %	98.0 ± 0.3	98.1 ± 0.3	97.9 ± 0.5
BMI *z*-score	2.4	2.5	2.2
Trunk fat (%)	46.1 ± 1.4	48.1 ± 1.7	43.9 ± 2.3
Total fat (kg)	38.6 ± 2.4	41.1 ± 3.6	35.2 ± 2.7
Percent fat (%)	43.9 ± 1.0	45.8^∗^ ± 1.2	41.8^∗^ ± 1.5
Total lean (kg)	46.0 ± 2.2	47.4 ± 3.4	44.2 ± 2.3
Energy intake (kcal/d)	2105	2031	1991
MetSyn (%)	8 (29%)	4	4

^∗^Indicate significant difference between diet groups (*P* < 0.05).

STAN: standard diet (55% calories from carbohydrate).

SPEC: specialized diet (43% calories from carbohydrate).

**Table 2:** Circulating 25OHD levels at baseline and five weeks in the presence or absence of MetSyn.

	Baseline	SE	Five weeks	SE
MetSyn	17.6	1.59	17.7	1.49
No MetSyn	19.1^∗^	0.93	21.3^∗^	0.94

^∗^Indicates significant difference between baseline and five weeks (*P* < 0.05).

Abbreviations: standard error (SE).

**Table 3:** The contribution of ΔOHD over the five weeks to MetSyn components represented by two models. Model A illustrates the independent contribution of ΔOHD to metabolic parameters and Model B presents the inclusion of both ΔOHD and PTH as independent variables.

	STAN				SPEC			
REG Model A	ΔOHD^a^		*P* value		ΔOHD^a^		*P* value	
Glucose (mg/dL)	−0.06 ± 0.43		0.88		−2.33 ± 1.01^∗^		0.04	
SI (×10^−4^ min^−^ *¹*/(*μ*IU/mL))	−0.04 ± 0.06		0.49		0.66 ± 0.26^∗^		0.02	
LDL (mg/dL)	−1.53 ± 0.60^∗^		0.02		0.99 ± 2.54		0.70	
HDL (mg/dL)	−0.33 ± 0.38		0.39		−0.47 ± 0.89		0.60	
Triglycerides (mg/dL)	0.67 ± 0.44		0.14		4.71 ± 3.46		0.19	
Trunk fat (kg)	0.18 ± 0.12		0.15		0.35 ± 0.35		0.34	

REG Model B	ΔOHD^a^	*P* value	PTH^a^	*P* value	ΔOHD^a^	*P* value	PTH^a^	*P* value

Glucose (mg/dL)	0.68 ± 0.36	0.07	0.29 ± 0.07^∗^	0.0007	−2.31 ± 1.04^∗^	0.05	−0.14 ± 0.26	0.60
SI (×10^−4^ min^−^ *¹*/(*μ*IU/mL))	−0.03 ± 0.07	0.69	0.00 ± 0.01	0.73	0.66 ± 0.22^∗^	0.01	−0.11 ± 0.05^∗^	0.04
LDL (mg/dL)	−1.84±0.70^∗^	0.02	−0.13 ± 0.15	0.38	1.10 ± 2.62	0.68	0.31 ± 0.55	0.58
Triglycerides (mg/dL)	0.11 ± 0.37	0.75	−0.27 ± 0.07^∗^	0.002	4.62 ± 3.62	0.23	−0.20 ± 0.85	0.81

^∗^Indicates significant difference (*P* < 0.05).

^a^Values represent beta coefficient and standard error.

Abbreviations: insulin sensitivity (SI), low-density lipoprotein (LDL), high-density lipoprotein (HDL), parathyroid hormone (PTH), and change in 25OHD concentration (ΔOHD).
